# Cytokine Imbalance as a Biomarker of Intervertebral Disk Degeneration

**DOI:** 10.3390/ijms24032360

**Published:** 2023-01-25

**Authors:** Natalia A. Shnayder, Azamat V. Ashhotov, Vera V. Trefilova, Zaitun A. Nurgaliev, Maxim A. Novitsky, Elena E. Vaiman, Marina M. Petrova, Regina F. Nasyrova

**Affiliations:** 1 Institute of Personalized Psychiatry and Neurology, Shared Core Facilities, V.M. Bekhterev National Medical Research Centre for Psychiatry and Neurology, 192019 Saint Petersburg, Russia; 2 Shared Core Facilities “Molecular and Cell Technologies”, V.F. Voino-Yasenetsky Krasnoyarsk State Medical University, 660022 Krasnoyarsk, Russia; 3 The Hospital for War Veterans, 193079 Saint Petersburg, Russia

**Keywords:** cytokines, cytokine status, disk degeneration, pathophysiology, biomarker, chronic inflammation

## Abstract

The intervertebral disk degeneration (IDD) and its associated conditions are an important problem in modern medicine. The onset of IDD may be in childhood and adolescence in patients with a genetic predisposition. IDD progresses with age, leading to spondylosis, spondylarthrosis, intervertebral disk herniation, and spinal stenosis. The purpose of this review is an attempt to summarize the data characterizing the patterns of production of pro-inflammatory and anti-inflammatory cytokines in IDD and to appreciate the prognostic value of cytokine imbalance as its biomarker. This narrative review demonstrates that the problem of evaluating the contribution of pro-inflammatory and anti-inflammatory cytokines to the maintenance or alteration of cytokine balance may be a new key to unlocking the mystery of IDD development and new therapeutic strategies for the treatment of IDD in the setting of acute and chronic inflammation. The presented data support the hypothesis that cytokine imbalance is one of the most important biomarkers of IDD.

## 1. Introduction

Intervertebral disk degeneration (IDD) is a multifactorial, chronic, recurrent disease that initially appears in the nucleus pulposus (NP) of the intervertebral disc (IVD), spreads to the annulus fibrosus (AF), then to other elements of the spinal motor segment (VMS), manifesting itself in certain conditions polymorphic (reflex, compression, compression-reflex and reflex-compression) neurological syndromes [1]. IDD is a pressing issue facing healthcare. The main manifestation of IDD is back pain. It is experienced by more than 85% of people over 35 years of age [2]. IDD-associated back pain is the leading cause of disability benefits in the social security system [3].

IVD is one of the most complex anatomical formations of the musculoskeletal system.

Functionally, it is a form of continuous cartilaginous joints, occupying an intermediate position between synchondrosis (sedentary strong bone adhesions) and true joints. The most important function of IVD is the leveling of the difference in loads and oscillatory movements on the spinal column, which is due to the peculiarities of its structural organization. Human IVD refers to avascular structures, in connection with this, a dosed load serves as an active stimulator of nutrient intake, the effect of which is not manifested in conditions of static postures and/or high stresses. During natural aging processes, the IVD structure undergoes significant changes in terms of the extracellular matrix and the state and density of NP and AF cell populations. The frequency of spondylosis and osteochondrosis, which represent the last stage of IDD, steadily increases with age and reaches 100% by the age of 80–90 [4].

IVD consists of two main functional blocks. The external block is AF, consisting mainly of collagen, and provides tensile strength, while the internal block is NP, which is rich in proteoglycans, and provides the hydrostatic properties of the IVD. Collagen types I and II make up 80% of the dry weight, with collagen type I predominating in AF and collagen type II in NP. Notochordal cells are the predominant cell population in human NP until they gradually disappear when the human reaches approximately 12 years of age [5]. IVD undergoes age-related changes earlier than many other tissues of the human body, which leads to histomorphological and functional changes [6]. The boundary between AF and NP becomes more and more clear as the organism grows. With aging and progressive IDD, NP is primarily affected. It becomes more fibrous and less elastic. Tiny concentric breaks appear in the outer part of the AF, from where they propagate into the NP. As a result of apoptosis, the amount of fibrous tissue increases, the composition and amount of proteoglycans change, and the number of cells changes [7].

While age-related changes in the IVD are normal, the process of IVD degeneration is a distinct pathological condition that includes structural failure and can occur at an increasing (accelerated) rate (premature aging, which leads to changes in the IVD microenvironment and promotes accelerated catabolism). Collagen type II fibers are replaced by collagen type I fibers in the interior of the AF and NP, and NP begins to accumulate yellow pigmentation, which also makes it less distinguishable from AF [6].

**Figure 1:**
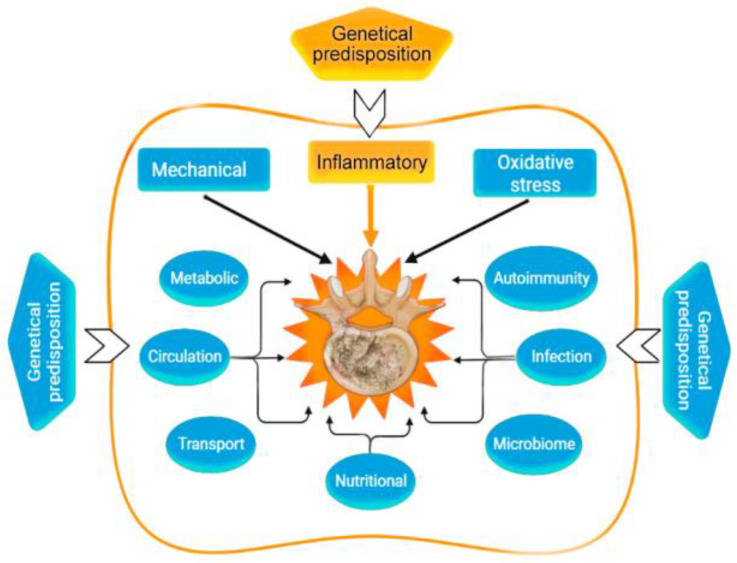
Risk factors of intervertebral disc degeneration.

Various factors play an important role in IDD (Figure 1).

The fibers in AF become more and more disoriented, and the network of elastin and collagen fibers gradually breaks down. Cells in NP undergo apoptosis and then necrosis; at the same time, they tend to over-proliferation. These degenerative cascades are frequent, and in adult IVD up to 50% of the cells may be necrotic [8].

The main mechanism of IDD is the loss of proteoglycans. These large molecules are broken down into smaller fragments that are lost from IVD tissue [9]. The consequence of this is a drop in osmotic pressure in the disc matrix and the loss of water molecules, which affects the mechanical properties of the IVD. Because degenerated IVDs contain less water and therefore have a poorer ability to withstand pressure, they bulge and lose height. The loss of proteoglycans also affects the movement of other molecules into and out of the IVD extracellular matrix.

Serum proteins and cytokines diffuse into the extracellular matrix, affect cells and accelerate the process of IDD [9]. The orientation, location, and types of collagen fibers are affected the most, but the total amount of collagen is less affected. Old collagen fibers are denatured, although new fibers are synthesized early in the degeneration process. Enzyme activity plays an important role in the process of denaturation and breakdown of collagen, fibronectin, and proteoglycans. Matrix metalloproteinases (MMPs) and cathepsins are the most important among others [10].

IDD is associated with damage to nearby structures such as ligaments, joints, and spinal muscles. This results in functional changes and greater susceptibility to injury. Due to overload, the degenerated IVD is located lower than normal, and the apophyseal joints must bear higher loads [10]. The consequence of this is osteoarthritis degeneration. The strength of the yellow ligaments decreases, which leads to their hypertrophy and protrusion of the ligaments into the spinal canal, followed by narrowing and compression of the neural structures [11].

There are many hypotheses for the development of IDD (Table 1).

Pain in IDD is complex and in many cases is a fair combination of structural and mechanical deformities, as well as the activity of inflammatory mediators. Often, spinal nerve roots are involved in the degenerative cascade, which causes chronic pain mainly due to their compression and partly due to the ingrowth of the smallest nerve endings into the degenerated IVD and their activation due to the constant release of inflammatory mediators [7].

The etiological role of genetic factors [15,19], smoking [28], infections [56,58], metabolic changes [34,36], impaired biomechanical load [25,26], reduced diffusion of nutrients through the endplate [32], and much more are discussed. One of the key factors in the development of IDD is a high level of synthesis of pro-inflammatory cytokines [46]. IDD is mediated by the release of large amounts of pro-inflammatory cytokines by NP cells, as well as by macrophages, neutrophils, and T- and B-lymphocytes. Synthesized cytokines induce a chain of pathophysiological reactions that lead to degeneration [39,67,68], oxidation [69,70], autophagy [71,72], aging [73,74], and apoptosis [69,71,75] of IVD cells.

The purpose of this narrative review is to summarize the data characterizing the patterns of pro-inflammatory and anti-inflammatory cytokines production in the development of IDD and to update knowledge about the predictive and protective significance of cytokine imbalance as a biomarker of IDD.

## 2. Pathogenetic Aspect of Inflammation in Intervertebral Disk Degeneration

There are many factors leading to a chronic inflammatory process in IVD. Studies on this topic have led to the formation of several hypotheses (Table 2).

## 3. Cytokines Alteration in Intervertebral Disk Degeneration

Cytokines, which consist of a family of proteins—interleukins (IL), lymphokines, monokines, interferons, and chemokines—are important components of the immune system (Table 3) [144]. They act in conjunction with specific cytokine inhibitors and soluble cytokine receptors to regulate the human immune response. Their physiological role in inflammation and pathological role in systemic inflammatory conditions are now well known. An imbalance in cytokine production or cytokine receptor expression and/or dysregulation of the cytokine process contributes to various pathological disorders, including IDD. Cytokines are classified as pro-inflammatory and anti-inflammatory. Time-dependent pro- and anti-inflammatory cytokine imbalance determines the outcome of the inflammatory response in the development of IDD. It should be clarified that the division of cytokines into pro- and anti-inflammatory is very arbitrary, since, depending on the conditions, a cytokine can behave as a pro- or anti-inflammatory cytokine (for example, IL-6). The number of cytokines, the nature of the activating signal, the nature of the target cell, the nature of the cytokines produced, the timing, the sequence of action of the cytokines, and even the experimental design are parameters that strongly influence the properties of the cytokines [144].

### 3.1. Pro-Inflammatory Cytokines

Pro-inflammatory cytokines play a central role in inflammatory diseases of infectious or non-infectious origin [49]. Pro-inflammatory cytokines are predominantly produced by activated macrophages and are involved in enhancing inflammatory responses [145]. These cytokines serve to contain and eliminate inflammatory lesions by activating local and systemic inflammatory responses. Pro-inflammatory cytokines can directly modulate cell activity in various IVD structures, including NP, AF, and extracellular matrix [2]. The main pro-inflammatory cytokines responsible for early responses are IL-1α, IL-1β, IL-6, and tumor necrosis factor-alpha (TNF-α). Other pro-inflammatory mediators include family members of IL-20, IL-33, leukemia inhibitory factor (LIF), interferon-gamma (IFN-γ), oncostatin M (OSM), ciliary neurotrophic factor (CNTF), transforming growth factor beta (TGF-β), granulocyte-macrophage colony-stimulating factor (GM-CSF), IL-11, IL-12, IL-17, IL-18, and a number of other chemokines that chemoattract inflammatory cells. These cytokines act as endogenous pyrogens (IL-1, IL-6, TNF-α), increase the synthesis of secondary mediators and pro-inflammatory cytokines by both macrophages and mesenchymal cells, stimulate the production of acute phase proteins, or attract inflammatory cells. IL-1β, TNF-α, IFN-γ, IL-12, and IL-18 are well characterized as pro-inflammatory cytokines [144].

#### 3.1.1. Interleukin 1β

IL-1β is produced by myeloid blood cells and pathogenic lymphocytes in autoimmune diseases [146,147] and degenerative [76] and metabolic diseases [148,149]. It is a key pro-inflammatory cytokine involved in the regulation of the innate immune response [150]. Apoptotic macrophages release IL-1 but not IL-6 or TNF-α, suggesting that in vivo macrophage apoptosis in IVD is the source of cytokine release. Ollmarker et al. [2] proved the ability of IL-1 to change the membrane potential and disrupt axonal transport in fibers. Thus, IL-1 promotes programmed cell death of IVD cells, activation of inflammation involving lymphocytes, macrophages, and neutrophils, and vasculogenesis and neurogenesis in degenerated IVD.

IL-1β can promote the expression of matrix metalloproteinases (MMPs), or zinc-containing and calcium-dependent endopeptidases in IVD [151]. Shi et al. [152] suggested that IL-1β can induce MMP-1 production. In another study, IL-1β stimulation caused a dramatic increase in MMP-1 and MMP-3 in human AF [101]. In addition, Zhan et al. [67] found that IL-1β stimulation increased the production of catabolic enzymes (MMP-10, MMP-9, and MMP-3), but decreased the expression of aggrecan and type II collagen. Fang et al. [102] demonstrated that IL-1β can induce the production of MMP-1, MMP-3, MMP-13, and ADAMTS-4. Moreover, mice without a natural inhibitor of IL-1R show a clear increase in MMP-3 and MMP-7 and exhibit similar features associated with human IDD [103].

IL-1β promotes a disintegrin and metalloproteinase with thrombospondin motifs (ADAMT) expression, which can regulate the production of ADAMTS-4 and ADAMTS-5 in IVD, thereby contributing to the loss of extracellular matrix and the development of IDD [76]. IL-1β is associated with stress-induced premature aging. Yang et al. [73] found that β-gal (β-galactosidase) levels were significantly elevated in IL-1β stimulated NPs. Li et al. [77] and Chen et al. [132] demonstrated that IL-1β promotes the progression of IDD with markedly increased expression of p16, p53, and SA-β-Gal.

Chondrocytes stimulated with IL-1β exhibit characteristics of aging phenotypes, which include increased SA-β-Gal activity, altered cell morphology, cell growth arrest, and telomere erosion. Mechanically, the expression of aging IVD phenotypes is partly associated with an increase in caveolin 1 and activation of p38 MAPK, as well as P53/P21/retinoblastoma pathways [143]. In addition, an increase in the number of senescent IVD cells has been found to decrease the self-renewal capacity of IVD cells and produce more extracellular matrix-degrading enzymes and inflammatory cytokines, resulting in a worsened IVD microenvironment [76].

IL-1β mediates apoptosis in NP and AF, a process that is closely correlated with IDD [76]. Wang et al. [75] suggested that IL-1β promotes the production of pro-apoptotic proteins, including cleaved caspase 3 (apoptosis coordinator enzyme) and Bax (apoptosis promoter protein), and reduces the production of anti-apoptotic content in IVD. Jiang et al. [118] found that IL-1β stimulation dramatically increased caspase-3 activity, cell apoptosis rate, and production of cleaved PARP (poly (ADP-ribose) polymerase), Bax, caspase-3, and cleaved caspase-3, but reduced the level of Bcl-2 (an apoptosis inhibitory protein) in IVD rats. Wang et al. [119] found that stimulation with IL-1β leads to a sharp increase in the rate of apoptosis in rat AF due to an increase in the activity of caspase-3, which is also inhibited by 17β-estradiol.

IL-1β is associated with pyroptosis, which is also detected by TUNEL staining (a method for detecting DNA fragmentation by labeling the 3′-hydroxyl ends in DNA double-strand breaks generated during apoptosis). Pyroptosis is a recently discovered form of inflammatory programmed cell death associated with IL-1β secretion. The process of pyroptosis is pro-inflammatory and is triggered by the NOD-like receptor family pyrin domain containing 3 (NLRP-3) inflammasome [152], which depends on the formation of oligomers of apoptosis-associated spotted proteins known as pyroptosomes [120]. In addition, pyroptosis has been found to be associated with P. acnes-mediated proinflammatory IDD. Elevated levels of NLRP3 (Nod-like receptor of the NALP family), the main component of the inflammasome type of the same name), IL-1β, caspase-5, caspase-1, and GSDMD (gastermin D, a tumor growth suppressor) were found in NP after co-cultivation with P. acnes [69].

Many factors have been found that can modulate NP cell proliferation, such as thymosin beta-4, IGF-1, and leptin. Similarly, IL-1β can also regulate IVD proliferation [76]. Wang et al. [125] found that IL-1β stimulation significantly suppressed IVD proliferation. In contrast, Li et al. [77] suggested that IL-1β drastically inhibits IVD cell proliferation and telomerase activity, and promotes G0/1 cell cycle arrest.

IL-1β dramatically increases intracellular reactive oxygen species in the IVD extracellular matrix in mice. Conversely, fullerol nanoparticles can prevent the IL-1β-mediated production of reactive oxygen species. Stimulation of bovine NPs by resveratrol can also inhibit IL-1β-induced oxidative stress [153]. In addition, Mathy-Hartert et al. [154] found that bovine chondrocytes incubated with IL-1β significantly reduced the production of superoxide dismutase, as well as catalase, which forms the first line of cellular defense against reactive oxygen species. This indicates that IL-1β may inhibit the antioxidant abilities of IVD.

Various studies have shown that neoinnervation and angiogenesis are dramatically increased in degenerative IVDs and positively correlate with the severity of IVD degeneration [142]. This indicates that neoinnervation and angiogenesis may play a significant role in the progression of IDD. Overexpression of vascular endothelial growth factor (VEGF), the most important pro-angiogenic factor, leads to IDD [139]. Similarly, overexpression of neurotrophic factors such as nerve growth factor (NGF) and brain-derived neurotrophic factor (BDNF) also accelerates IDD [140]. IL-1β plays a role in enhancing the expression of VEGF, NGF, and BDNF in IDD. Under hypoxic conditions, IL-1β increased VEGF production in IVD cells, while anti-IL-1β antibody treatment decreased VEGF production. The levels of VEGF, BDNF, and NGF in degenerated IVDs were markedly increased by IL-1β stimulation, and the concentration of IL-1β shows a positive correlation with the level of expression of these factors. Human IL-1β-stimulated AF also shows a significant increase in NGF and BDNF [76]. Conversely, high molecular weight hyaluronic acid hydrogels have been found to reduce IL-1β-mediated NGF and BDNF by inhibiting the IL-1R1/MyD88 pathway in bovine NPs [141]. Together, IL-1β promotes angiogenesis and neoinnervation by inducing the production of VEGF, NGF, and BDNF [76], which may play a role in the development of IDD.

#### 3.1.2. Interleukin 2

IL-2 is mainly produced by mature T cells and is involved in the development of T cells and B cells since it can function as a growth factor for them [155]. Although IL-2 plays an important role in inflammatory processes, its expression level did not differ significantly in patients with acute or chronic low back pain, or even in asymptomatic subjects. Moreover, IL-2 levels were not associated with visual analog scale scores in either acute or chronic low back pain [156]. In a study by Weber et al. [157] measuring levels of various cytokines in low back pain, serum IL-2 levels in patients with low back pain were significantly lower than in controls, similar to other factors including IL-6, IL-4, and MMP-1. The SNV (−)330T>G of the *IL2* gene was significantly associated with IDD and occurred twice as often in patients than in controls. In addition, the *IL2* haplotype (“GT”) was also significantly associated with IDD [158]. It should be recognized that the role of IL-2 in the development of IDD continues to be studied, and its role in this disease is not yet clear.

#### 3.1.3. Interleukin 8

IL-8 is secreted predominantly in response to an antigen by macrophages, T-lymphocytes, neutrophils, and other cells; IL-8 is also the most potent human chemokine [144]. IL-8 causes hyperalgesia by causing the local production of sympathetic amines that increase the sensitivity of nociceptors [158]. In IVD, oxidative/nitrosative stress and damage caused by mechanical stress lead to an increase in IL-8 levels [109]. Interestingly, not only local IL-8 expression but also serum expression and cerebrospinal fluid concentration depend on radicular pain [159]. Patients undergoing multi-level IVD surgery also demonstrated elevated levels of IL-8 compared to single-level surgery patients [85]. IL-8 was significantly elevated in patients with chronic low back pain who report pain and disability compared to people without pain with or without IDD. IL-8 and its murine homolog CXCL5 (LIX) were also upregulated in IDD compared to healthy controls in humans and mice, respectively [85]. A comparison between tissue from degenerative and herniated IVDs showed that IL-8 levels were higher in IDD, leading to the suggestion that elevated IL-8 levels may contribute to the more severe back pain seen in IDD. The results of a study by Krock et al. [86] point to a role for IL-8 in low back pain, demonstrating an increase in: (a) cerebrospinal fluid in patients with low back pain compared to pain-free subjects with and without IDD; (b) in IDD obtained from patients with low back pain compared to a control group without IDD. Both mechanisms that initiate and maintain IDD, namely, mechanical stress and sterile inflammation, have been found to regulate IL-8 in IVD.

Unfavorable mechanical stress on human NP and AF cells and acute ex vivo mechanical trauma to human IVD induce an increase in IL-8 secretion [111]. An increase in IL-8 levels after sterile IVD inflammation has been shown, including TLR2 NP activation [160], TNFα treatment of AF cells [161], IL-1β treatment of NP cells [162], and TLR2 activation and TNFα and IL-1β treatment of mixed IVD cells [163]. The classically described role of IL-8 is to induce chemotaxis and activate neutrophils, as well as to stimulate vascularization. Infiltrating neutrophils have been found in degenerating IVDs, where they likely contribute to a pro-inflammatory and catabolic environment [106]. However, in a study by Phillips et al. [107], exposure of NP cells to IL-8 did not alter the expression of genes associated with IDD, including aggrecan, MMP-3, or MMP-13. Thus, while IDD controls pathways that likely activate IL-8, the effect of IL-8 on IDD requires further study [86].

#### 3.1.4. Interleukin 12

IL-12 is a cytokine whose main role is to connect innate and adaptive immunity [155]. It is secreted predominantly by macrophages and dendritic cells in response to components of the bacterial cell wall. IL-12 stimulates proliferation, and also activates and increases the cytotoxicity of natural killer (NK-cells) and T cells, promoting the differentiation of the latter into Th1 [144]. Together with other immune-related factors and molecules, this cytokine also plays a role in IDD. As with IFN-γ, IL-12 levels were higher in herniated IVD fragments [161]. It is also known to induce the secretion of IFN-γ and TNF-α and has a synergistic effect with IL-18 [144]. IL-12 mainly functions in conjunction with other cytokines such as IFN-γ, and the levels of these cytokines differ between IVD and IDD hernia fragments. Accordingly, both IL-12 and IFN-γ, as well as other cytokines (IL-4, IL-6), showed higher levels in IVD hernia fragments although, at the same time, expression levels of these cytokines did not differ much between NP and AF of healthy IVDs obtained at autopsy [164].

#### 3.1.5. Interleukin 17 

IL-17 is secreted by helper lymphocytes 17 (Th17) and stimulates macrophages to secrete pro-inflammatory cytokines [144]. IL-17 accelerates the development of IDD by promoting the degradation of the extracellular matrix, enhancing the inflammatory response, inducing neo-angiogenesis, and inhibiting autophagy and NP cell proliferation. Targeting IL-17 represents a new and promising approach to therapeutic intervention in IDD [90].

High levels of IL-17A have been associated with IDD and IVD herniation, and IL-17A is considered a critical factor in IVD pathology [91,92]. In addition, a rat hanging or pierced tail model showed IL-17A expression in NP cells and AF cells with IDD or IVD tissue damage [115]. In an IVD hernia in which the NP herniated into a herniated AF, compared with healthy controls, elevated levels of Th17-lymphocytes and IL-17A correlated with the intensity of pain in patients with sciatica, suggesting that AF rupture and NP herniation are initiators of the autoimmune response to rupture lumbar IVD [91]. High levels of IL-6, IL-17A, and TNFα were observed in the serum of patients with lumbar radiculopathy compared with the group with neuropathic pain, and Th17 was higher in the venous blood of patients with lumbar radiculopathy compared with the group with neuropathic pain [165]. In addition, IL-23 [93] and IL-21 [166] have been reported to be cytokines that stimulate Th17 to produce IL-17A in IDD or IVD hernia. IL-23 expression was significantly increased in human lactate dehydrogenase tissues and showed a significant positive correlation between IL-23 and IL-17A expression. Therefore, the canonical inflammation-associated IL-23/IL-17A signaling axis may play a critical role in IDD [94]. Moreover, as an illustration of the correlation between IL-17A and IL-21, patients with lumbar IVD hernia had significantly higher serum levels of IL-21 and IL-17 than healthy controls [167].

Th17-associated cytokines such as IL-17A, IL-22, and TNFα upregulated the expression of CC chemokine ligand (CCL) 20 and its only receptor, CC chemokine ligand-receptor (CCR) 6, in human keratinocytes [168] Th17 cells predominantly express CCR-6 and produce CCL-20 as their ligand in models of rheumatoid arthritis [169]. CCR-6 is specifically expressed on the surface of Th17 cells and is associated with Th17 infiltration [170]. Human T-cell studies have shown that CD4+CD45RO+CCR6+ cells contain and secrete much more IL-17A mRNA and more IL-17 protein than CD4+CD45RO+CCR6- cells [171]. Studies analyzing CCL-20-CC-6 in disk tissues in patients with IVD disease have shown that IL-17A-producing cells (CD4+IL-17A+ and CD4+CCR6+) appear in NP tissues upon AF rupture. In addition, these studies have shown that NP cells can produce abundant CCL-20 and that the Th17-associated cytokines (IL-17A and TNFα) can enhance CCL-20 production [112]. TNFα stimulation promotes overexpression of the *CCL20* gene in IDD cells [69]. In an in vivo study, expression levels of CCL-20, IL-17A, and CCR6 in IVD tissues were dramatically elevated compared to control and IVD needle-piercing groups in an IDD needle-piercing model. NP tissue was transplanted onto the nerve root [113]. Analysis of the correlation between chemokines and inflammatory cytokine gene expression in humans showed a significant correlation between CCR-6 and IL-17A expression in both IVD tissues and blood samples [172]. These studies indicate that IL-17A-producing cells may be involved in IVD tissue degeneration through interaction with the CCL20/CCR6 system in vivo [91].

In an in vitro study, IL-17A treatment of IVD-derived NP cells showed that IL-17A inhibited cell proliferation and extracellular matrix synthesis [131]. In addition, treatment with IL-17A and anti-IL-17A neutralizing antibodies caused a significant decrease in the response of IL-6, COX-2, MMP-3, and MMP-13. Small molecule compounds identified as inhibitors by binding to the IL-17A-binding region of IL-17R using an in silico assay revealed effects similar to the evaluation of an IL-17A neutralizing antibody [92]. According to these studies, NP cells have an IL-17A receptor (IL-17R) on their cell surface, IL-17A can influence intracellular responses by forming the IL-17A/IL-17R complex, and IL-17A signaling is involved in the development of IDD.

The main downstream IL-17A signaling pathway is the NF-κB pathway [173]. The NF-κB 1 (Act1) activator contains a SEFIR domain and a TNF receptor-associated factor-6 (TRAF-6) binding motif. After IL-17A binds to IL-17R (IL-17RA/IL-17RC), Act1 interacts with the IL-17A receptor via the SEFIR domain, and TRAF-6 interacts with the TRAF-6 binding motif of Act1; subsequently, TRAF6 activates β-activated transforming growth factor kinase (TAK)1 and the NF-κB kinase inhibitor complex (IKK), consisting of IKKα, IKKβ, and IKKγ, and then NF-κB is activated [174]. Since IL-17A alone has been reported to be insufficient for strong NF-κB activation, IL-17A interacts with other cytokines such as TNF-α to stimulate NF-κB and enhance the stabilization of pro-inflammatory cytokine and chemokine mRNA expression [173]. Moreover, IL-17A–IL-17R–TRAF-6 can promote activation of the mitogen-activated protein kinase (MAPK) and the AP-1 (activator protein-1 transcription factor pathway) pathway [175]. IL-17A stimulation recruits TRAF-4, which competitively binds to the same TRAF6 binding site on Act-1 [176]. The Act1-TRAF-4 interaction specifically directs the activation of the MEKK3-MEK5-ERK5 cascade and leads to the activation of the MAPK pathway [177]. In addition, TRAF3 binds directly to IL-17RA and prevents IL-17RA-Act1-TRAF-6 interaction [178].

IL-17A significantly enhances the synthesis of COX-2 and prostaglandin-E2 (PGE-2) through activation of the MAPK/AP-1 pathway in NP cells, leading to activation of the inflammatory response in IDD. IL-17A, which induces COX-2 expression and increases PGE-2 production using AP-1-dependent mechanisms, works through parallel signaling cascades including p38/c-Fos and JNK/c-Jun (p38 kinase, c-Jun N -terminal kinase (JNK)). Increased expression of the c-fos gene in NP cells leads to the progression of IVD degeneration [95]. IL-17A also greatly enhances the phosphorylation of c-Jun, which is not only a transcription factor in MAPK but also a JNK chain. Additionally, IL-17A can induce nuclear translocation of c-Jun and c-Fos, which can be stopped by some JNK and p-38 inhibitors [179].

Similarly, IL-17A has been reported to interact with many cytokines such as TNFα or chemokines in IVD cells [92]. As mentioned above, IL-6 is required for Th17 differentiation to produce IL-17A, and it has also been expressed through IL-17A signaling in IVD cells via MAPK pathways [92] In addition, IL-1β has been reported to induce IL-17A expression with IDD [69]. In addition, the TNF receptors TNFR-1 and TNFR-2 are involved in the IL-17A response in IDD. TNFR-1 can be activated by either tmTNF-α (transmembrane TNFα) or sTNF-α (soluble form), while TNFR-2 is activated primarily by sTNF-α. Upon stimulation with tmTNF-α and sTNF-α, the TNFR-1/silencer of death domains (SODD) complex releases the inhibitory protein SODD, and TNFR-1 is activated. TNFR-1 then binds to the TNF receptor-associated death domain, recruiting other adapter proteins, including TNF receptor-associated factor 2 (TRAF-2), receptor-interacting protein-1 (RIP-1), and cellular inhibitor of apoptosis protein (cIAP) 1, resulting in the formation of complex I, which signals via the NF-κB or MAPK pathways to activate p65 or AP-1 [69]. TNFR-2 recruits TRAF-3, TRAF-2, cIAP1/2, and TRAF-1 to form a complex that also activates NF-κB, AP-1, and ERK and, consequently, activates PI3K/AKT [69]. This regulation concerns many IL-17A responses in IVD cells. In addition, these pathways regulate the production of pro-inflammatory mediators such as TNF-α, IL-1β, or IL-6, and these mediators can again recruit Th17 cells that produce IL-17A. Indeed, when a factor that inhibits TNF-α-TNFR-1 signaling was downregulated in mouse models of IVD degeneration, IL-17A expression was significantly increased by TNFα stimulating NF-κB and MAPK pathways via TNF-α receptors. TNF-α can directly stimulate IVD cells and affects IL-17A signaling. Hence, this leads to the progression of IDD [96].

Peroxisome proliferator-activated receptor-γ (PPAR-γ) activators have an anti-inflammatory and antidegenerative role in osteoarthritis and rheumatoid arthritis. PPAR-γ was downregulated in both the nucleus pulposus tissue of an IDD patient and cultured nucleus pulposus cells stimulated with IL-17 [68].

IL-17A can inhibit autophagy in human IDD NP cells by activating the PI3K/Akt/Bcl-2 signaling pathway (the classic anti-apoptosis pathway in cells). Based on the protective role of autophagy in IDD, it can be concluded that IL-17A promotes the development of IDD by inhibiting autophagy [72].

#### 3.1.6. Interleukin 18

IL-18, a member of the IL-1 superfamily with a structure similar to IL-1β, is a highly regulated inflammatory cytokine that is cleaved by the intracellular protease caspase-1 to form a biologically active molecule. IL-18 has been reported to be elevated in inflammatory diseases and conditions such as type 2 diabetes mellitus, obesity, Alzheimer’s disease, and coronary heart disease [180]. IL-18-mediated activation of T cells and natural killer cells leads to the secretion of IFN-γ, which, in turn, activates macrophages that secrete the cytokines TNF-α and IL-1, which cause increased degradation of the matrix as both directly and through the activation of proteinases like MMPs [181]. These molecules have been found to be secreted by cells of degenerated and herniated IVDs and therefore play a role in IDD [82].

IL-18 increases the production of reactive oxygen species in cells by activating caspase-1 and the inflammasome system, which leads to further production of IL-18 and neuronal apoptosis [121]. Zhang et al. [122] demonstrated that the downregulation of IL-18 by caspase-3/9 can reduce NP cell death as well as the imbalance between extracellular matrix catabolism and anabolism in degenerative IVD.

It has been shown that IL-18 can increase levels of the anti-apoptotic proteins B-cell lymphoma 2 (BCL-2) and B-cell lymphoma-extra-large (BCL-XL) [123]. A study by Tang et al. [70] showed that IL-18 released from pyroptotic NP cells causes degeneration of surrounding normal NPs, thereby accelerating IDD. IL-18 can also affect vascular endothelial cells of the endplate, hence changing the environment around NP cells, AF cells, and endplate chondrocytes. The main pathological changes associated with IDD include cartilage end plate degeneration and NP senescence or apoptosis. Endplate cartilage calcification is the main cause of endplate degeneration [39]. IL-18 can also induce inflammatory responses in synoviocytes and chondrocytes and increase the expression of inflammatory factors such as TNF-α, PGE2, and COX-2. Thus, it contributes to cartilage degeneration and osteoarthritis [83]. IL-18 degrades the IVD matrix and is elevated in the sera of patients with IDD. In another study, IL-18 upregulated MMP-13 expression and suppressed the expression of anabolic factors such as type II collagen and SOX-6 in human NPs [105].

The *IL18RAP* gene encodes a protein that is an additional subunit of the heterodimeric receptor for the proinflammatory cytokine interleukin 18 (IL18) [182]. This protein enhances the IL-18 binding activity of the IL-18 receptor and plays a role in IL-18 signaling. SNVs of the *IL18RAP* gene may play a key role in IDD. The CT genotype of SNV rs917997 had a protective effect in women and patients who did not drink alcohol in anamnesis. This study is preliminary and further studies involving a larger population may provide a better idea [84].

#### 3.1.7. Tumor Necrosis Factor Alpha

TNF-α is a cytokine synthesized by many cells of the body, which is a type II transmembrane protein in the form of a stable homotrimeric structure. It exerts its multiple effects through the TNF receptor superfamily, which contains more than 20 structurally related transmembrane proteins, leading to a wide range of cellular responses. The TNF receptor superfamily can be divided into two functional types, depending on whether the intracellular region contains a death domain [183]. Receptors are represented by transmembrane proteins, which interact with the trimers of inducer ligands with their extracellular regions. It is the common property of the two types of receptors. The interaction between the receptor and the ligand leads to the formation of clusters of receptor molecules and the binding of their intracellular regions to adapters. The adapter, after binding to the receptor, interacts with the effectors [183,184]. TNF receptors containing death domains are more commonly known as death receptors [183]. The biological functions of TNF-α are mediated by its two main receptors: tumor necrosis factor receptor 1 (TNFR-1 or p55) and tumor necrosis factor receptor 2 (TNFR-2 or p75). TNFR-1 activation initiates inflammatory, apoptotic, and degenerative cascades, while TNF-α signaling through TNFR2 is anti-inflammatory and cytoprotective. This leads to the induction of proliferation, differentiation, angiogenesis, and tissue repair [144]. TNF-α is also an important pro-inflammatory cytokine produced by both AF, NP cells, and the extracellular matrix [76].

TNF-α enhances COX-2 expression in IVD cells and also increases the production of PGE-2 (regulating the activity of various signaling pathways through the G-protein family of prostaglandin receptors), which stimulates the TNF-α-PGE2 Wnt signaling pathway through the EP3 receptor. It has also been found that it is possible to control the expression of the Wnt signaling pathway through nuclear factor kappa-B (NF-κB). TNF-α activates p65, JNK, and p38 regions of the MAPK signaling pathway in NP cells in IVD. At the same time, stimulation of proliferative processes through TNF-α depends on the interaction of the NF-κB, JNK, and p38 signaling pathways. Among other things, it was found that short-term exposure to TNF-α stimulates proliferative processes through the MAPK pathway without the involvement of the extracellular signal-regulated kinase (Erk)1/2 region [185].

The stress-activated protein kinase cascade (SAPK signaling pathway) has an inhibitory effect on cell growth and inflammation activation in IVD through the phosphorylation of c-Myc and Elk-1 proteins. The result of the activation of the SAPK signaling pathway is the activation of apoptotic processes in IVD cell structures. In addition, activating this intracellular TNF-α cascade leads to damage to the myelin sheath of nerve fibers and increased sensitivity to pain. Iragashi et al. [2] showed that the addition of TNF-α to AF and NP cell culture induces the death of the latter; however, the introduction of monoclonal antibodies to TNF-α inhibits the process of cell apoptosis.

TNF-α can induce the production of various pro-inflammatory cytokines in IVD. TNF-α stimulation promotes the production of IL-8 and IL-6 in human AF. Upon stimulation with TNF-α, the level of substance P (SP) increased, which subsequently induced the expression of IL-1β, IL-6, and IL-8. TNF-α can also stimulate the synthesis of reactive oxygen species to produce IL-17, which is associated with the severity of IVD degeneration. In addition, in AF and NP cells in IDD surgery patients, both IL-17 and TNF-α can induce the secretion of inflammatory mediators, including IL-6, NO, and PGE2. IL-17 and TNF-α also increase the level of intercellular adhesion molecules (ICAM-1) in these cells [186]; and vice versa, IL-38 significantly reduced TNF-α stimulated expression of IL-1β, COX-2, and IL-6 in human NP cells. TNF-α can also modulate the production of various chemokines in IVD [69]. In a study, Liu et al. [187] found that the level of expression of the *CCL3*, *CCL20*, *CXCL2,* and *CXCL5* genes increased in IVD cells upon TNF-α stimulation. Consistent with this, TNF-α treated IVDs markedly increase CCL5 production, which is strongly associated with IDD severity and back pain [188]. In addition, Wang et al. [189] demonstrated that CCL-3, which also closely correlates with IDD severity, is significantly induced by the NF-κB and MAPK signaling pathways during TNF-α exposure. In addition, exposure to TNF-α on human AF cells showed a dramatic upregulation of CCL-2 expression [190].

Activation or suppression of nicotinamide phosphoribosyltransferase (NAMPT) activity controls TNF-α-induced destruction of the IVD extracellular matrix through the regulation of NLRP3 inflammatory activity. At the same time, melatonin can inhibit TNF-α-mediated destruction of the IVD extracellular matrix by reducing the activity of the NLRP3 inflammasome in NP cells [80].

Additionally, TNF-α is closely associated with mechanical stress on the IVD. Mechanical loading can trigger TNF-α expression and histological changes in IVD. TNF-α can enter healthy IVDs under dynamic mechanical stress on the VMS, promoting the production of other pro-inflammatory cytokines and altering the mechanical behavior of the IVD. In general, TNF-α is believed to be involved in increased inflammatory responses in the development of IDD [76].

TNF-α can stimulate the expression of multiple MMPs and ADAMTS. TNF-α induces the expression of these enzymes mainly through the NF-κB/MAPK signaling pathways. Thus, TNF-α dramatically increases the production of MMP-1, MMP-3, MMP-13, ADAMTS-4, and ADAMTS-5 in HNPC ex vivo, which leads to the degradation of aggrecan and collagen in IVD. Yang et al. [104] found that TNF-α stimulation markedly increased MMP-3 and ADAMTS-5 levels, while type II collagen levels decreased. Li et al. [77] demonstrated that TNF-α significantly increases the expression of MMP-13, MMP-3, and ADAMTS-4 proteins and genes, but reduces the production of type II collagen and aggrecan. Wang et al. [78] considered that TNF-α is critical for maintaining ADAMTS7 levels during inflammation in the NP. TNF-α treatment can inhibit the expression of various types of collagene, aggrecan, and fibromodulin, and increase the production of MMPs as well as pain-associated molecular nerve growth factor (NGF). Moreover, when AF cells were stimulated with TNF-α, MMP-1 production was also increased. In addition, exposure of NP cells to TNF-α increases the level of PHD2, which can interact with NF-κB to induce transcription of ADAMTS-5, MMP-13, and MMP-3. Similarly, PHD-3 is also involved in NF-κB activation and TNF-α-induced production of ADAMTS-5 and MMP-13 [76]. In addition, TNF-α induces Wnt5a expression, increasing extracellular matrix SOX-9-dependently by activating JNK-AP1 signaling (JunB) and inhibiting TNF-α mediated production of MMPs by NF-κB [79].

Li et al. [77] demonstrated that TNF-α treatment of IVD cells markedly increased SA-β-Gal activity, increased production of aging markers (p53 and p16), and increased G0/1 cell cycle arrest. Xie et al. [74] found that TNF-α enhances NP cell senescence, as evidenced by an increase in SA-β-Gal activity as well as the production of aging markers (p53 and p16). They found that estrogen protein-1 can suppress the effect of TNF-α on the aging of these cells.

TNF-α also caused cellular senescence in cartilage endplate stem cells (CESCs), while rapamycin-mediated autophagy prevented TNF-α mediated cellular senescence in CESCs [133]. Li et al. [134] found that 17-beta-estradiol can alleviate the TNF-α-mediated senescence of NP cells in rats by interacting with the ROS/NF-kB pathway. In addition, treatment with 17-beta-estradiol significantly increased cell proliferation efficiency but decreased SA-β-Gal activity and aging markers (p16 and p53) produced in TNF-α-stimulated NP cells. Moreover, TUG-1 LncRNA silencing could protect human NP cells from TNF-α mediated senescence by inhibiting the Wnt/β-catenin pathway, which could be the basis for future IDD therapy. Overexpression of zinc metallopeptidase STE24, which is associated with cell aging and premature aging, suppressed the aging effects of TGFβ/NF-κB in NP cells when treated with TNF-α [135].

TNF-α binds to TNF receptors, controlling the JNK/ERK-MAPK and NF-κB signaling pathways in NP cells in IDD, activating proapoptotic protein and downregulating antiapoptotic protein, thereby leading to cellular apoptosis. Yu et al. [116] found that TNF-α stimulation of IVD cells markedly increased caspase-3 activity, degree of apoptosis, Bcl-2, caspase-3 and Bax production, and NF-κB pathway activity. In both degenerative human AF and NP isolated from patients undergoing spinal surgery, TNF-α markedly increases the rate of apoptosis as well as the expression of caspase 3 and p53. Similarly, when rabbit IVD cells are exposed to TNF-α, many IVD cells undergo apoptosis and exhibit the related morphological features of IDD [76]. Moreover, reactive oxygen species can also mediate the pyroptosis of IVD cells via the NLRP-3/PYCARD pathway and downregulate by enhancing autophagy and nuclear erythroid-like transcription factor 2 (NFE2L-2) [72].

When human NP cells were exposed to TNF-α, the number and viability of these cells were significantly increased, and the levels of cyclin B1 were markedly increased, indicating increased cell proliferation. Chen et al. [126] demonstrated that TNF-α led to apoptosis of some IVD at an early stage, and then promoted the proliferation of surviving cells. Conversely, Lin et al. [127] showed that TNF-α treatment dramatically inhibited human NP cell viability and increased IL-1β levels in a time-dependent manner. Li et al. [77] demonstrated that TNF-α significantly reduced IVD cell proliferation and telomerase activity and promoted G0/1 cell cycle arrest. In addition, Cheng et al. [117] found that stimulation with a relatively high concentration of TNF-α (50–200 ng/mL) induces mesenchymal stem cell apoptosis. Conversely, a low concentration (0.1–10 ng/mL) enhanced the migration and proliferation of NP but suppressed their differentiation.

In terms of underlying mechanisms, TNF-α-induced human NP cell proliferation is associated with c-Jun N-terminal kinases (JNK), NF-κB, and p38 MAPK signaling pathways. Notch signaling promotes IVD cell proliferation [128]. Wang et al. [129] demonstrated that stimulation of IVD cells with TNF-α increased the production of Notch-1 and Notch-2 receptors, ligands, and target genes in rat IVD. Moreover, Notch-2 levels in IVD degenerative tissues are higher than in non-degenerative tissues. Chen et al. [130] found that acute exposure to TNF-α enhances NP cell proliferation by activating the UPR/XBP1 pathway. In addition, TUG-1 LncRNA silencing also promotes cell proliferation and protects human NP cells from TNF-α-mediated apoptosis by inhibiting the Wnt/β-catenin pathway [76].

The exact function of autophagy in IDD remains controversial and requires further study. Autophagy can play both a protective role and cause the progression of IDD. The relationship between autophagy and TNF-α in IVD cells has received a lot of attention. Compared to healthy IVD, levels of proteins associated with autophagy, including beclin 1, cathepsin B, presenilin 1, Autophagy-related protein (ATG)-8, and ATG-12 (ubiquitin-like proteins), are markedly elevated in IDD [137]. Furthermore, exposure of TNF-α on human AF cells dramatically increases the expression of autophagy-associated proteins, including p62, damage-regulated autophagy modulator 1, beta-transducine-repeat protein interacting with phosphoInositides (WIPI)-49, and serine/threonine-protein kinase (PIM)-2 [137]. This result indicates that TNF-α is an important promoter of AF cell autophagy. In addition, acute exposure to TNF-α triggered protein kinase, RNA-like kinase ER/eukaryotic translation initiation factor 2α (PERK/eIF2α), unfolded protein response, and activation of autophagy, while interference with the PERK/eIF2α pathway suppressed autophagy in IVD [136].

#### 3.1.8. Interferons

IFN-γ is a soluble cytokine that is predominantly released by T-helper type 1 (Th-1), cytotoxic T lymphocytes, and NK-cells [144]. Due to the well-known role of IFN-γ in arthritis, this cytokine has also become an object of attention in the study of the mechanisms of IDD. In particular, an increase in its level was confirmed in IDD and IVD hernias [2].

During the development of IDD and disc herniation, IFN-γ is one of the inflammatory components that is activated in NP and affects tissue-specific macrophages in NP [191]. When IFN-γ is produced by Th-1-lymphocytes in IVD, it is involved in the activation of macrophages, which can be considered an immune response to NP herniation [164]. Moreover, IFN-γ also plays a role in the pathogenesis of neuropathic pain [192]. Since IFN-γ is activated during neuroinflammation and also affects nociceptive neurons, any structural change in its gene that results in higher expression could possibly play a role in the pathogenesis of the disease. Therefore, it was found that some specific SNVs of the *IFNG* gene affect the level of its expression [191].

A study by Teodorczyk-Injeyan et al. [156] was shown higher levels of IFN-γ in acute low back pain compared to chronic low back pain or in healthy subjects. Interestingly, IFN-γ levels did not differ significantly between people with chronic low back pain and asymptomatic people. The expression level of IFN-γ was associated with an increase in pain scores on a visual analog scale. Accordingly, higher levels of IFN-γ were found in patients who did not respond to surgery. Kamieniak et al. [192] found that IFN-γ did not change significantly during spinal cord stimulation, as an option to reduce pain.

IFN-γ levels have been associated with acute low back pain, although its association with chronic low back pain in IDD patients was not significant [156]. In the study by Hanaei et al. [193], SNV rs2069705 of the *IFNO* gene did not show a significant association with postoperative pain reduction. In the study by Moen et al. [191], allele A of rs2069705 was associated with a higher Oswestry Disability Index (ODI), because patients with AA and GA genotypes had significantly higher ODI scores. In addition, genotypes AG and GG rs2069718 were associated with higher ODI rates in the Norwegian population.

Interestingly, IFN-β1 (similar to IL-1β and TNF-α) may promote autophagy for certain cell types [138], including with IDD [138].

Results of Sadowska et al. [110] demonstrated that IFN-α1, IFN-α8, and IFN-β1 are internally correlated and expressed at significantly higher levels than TNF-α, but at similar levels to IL-6 and IL-8. They also showed a negative correlation of IFN-α1 with IDD grade and increased expression in lumbar samples compared to cervical samples.

**Figure 2:**
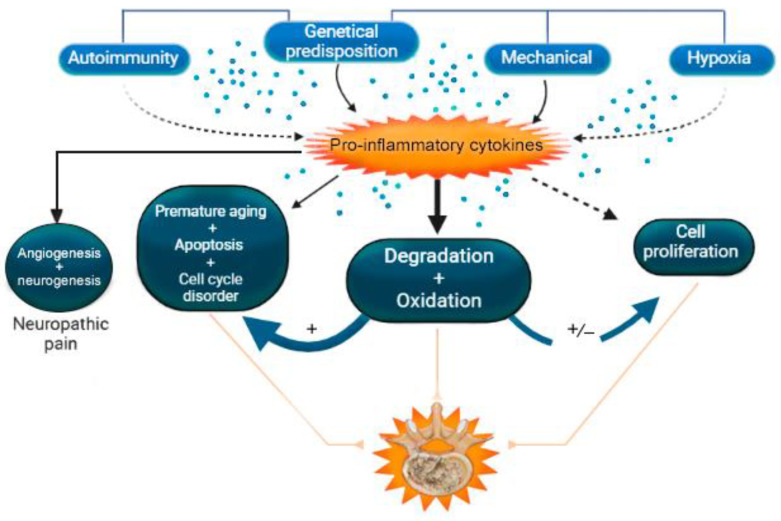
Effects of pro-inflammatory cytokines in intervertebral disk degeneration (IDD). Note: (+)—relationship between the mechanisms of IDD development; (+/−)—possible relationship between the mechanisms of IDD development.

A summary of the role of pro-inflammatory cytokines in IDD is presented in Table 4 and Figure 2.

### 3.2. Anti-Inflammatory Cytokines

Anti-inflammatory (immunosuppressive) cytokines are a series of immunoregulatory molecules that control the response of pro-inflammatory cytokines. Anti-inflammatory cytokines act in conjunction with specific inhibitors of pro-inflammatory cytokines and soluble cytokine receptors to regulate the human immune response. Major anti-inflammatory cytokines include an antagonist of the interleukin 1 receptor (IL-1Ra), IL-4, IL-6, IL-10, IL-11, IL-13, and TGF-β. Specific cytokine receptors for IL-1, TNFα, and IL-18 also function as inhibitors of pro-inflammatory cytokines: IL-1Ra as IL-1α and IL-1β antagonist; IL-18 binding protein (IL-18BP) as IL-18. Several newly discovered cytokines, such as IL-33, IL-35, and IL-37, are also involved in the regulation of neuronal and neuroglia function. Anti-inflammatory cytokines, in particular IL-10, inhibit the synthesis of pro-inflammatory cytokines and the expression of adhesion molecules, increasing the level of specific cytokine inhibitors. IL-1Ra, IL-4, IL-6, and interleukin 10 (IL-10) are well characterized as anti-inflammatory cytokines [144].

#### 3.2.1. Interleukin 4

IL-4 is produced by activated T-helper (Th) lymphocytes, mainly Th2 lymphocytes, natural killer T cells (NK cells), mast cells, and basophils. Its role is to promote the differentiation of Th into Th2 lymphocytes, as well as to increase their cytotoxicity [144].

Te Velde et al. [194] first demonstrated the anti-inflammatory properties of IL-4 in the treatment of IDD. This cytokine was able to inhibit lipopolysaccharide (LPS) or IFN-γ and induced the production of IL-1β and TNF-α a in monocytes. In chondrocytes, IL-4 has been shown to effectively inhibit IL-1α- and TNF-α-induced pathways [195]. The anti-inflammatory effects of IL-4 have also been demonstrated in vivo. In a rat model of bacterial cell wall-induced arthritis, daily administration of recombinant mouse IL-4 was able to reduce the arthritic index (erythema, edema, and deformity) and reduce the number of inflammatory cells in the peripheral blood and peritoneal cavity [196]. In an experimental osteoarthritis model, daily intra-articular injection of recombinant rat IL-4 showed a reduction in histopathological scores compared to untreated knee joints after 6 weeks [197].

The introduction of IL-4 into the joints using viral vectors reduced cartilage destruction, aggrecan degradation, and MMP activity in a mouse arthritis model [198]. Articular delivery of dexamethasone with recombinant mouse IL-4 fused to the variable chain single clonal antibody F8 (F8-IL4) was also able to reduce arthritic scores, inflammatory cells, and cytokines in the arthritic paw in a mouse model [199]. Taken together, these studies suggest that IL-4 administration may be a promising therapeutic strategy that will reduce IVD inflammation and its rate of degeneration [200].

Lowenthal et al. [201] found that most IVD cell lines express IL-4, but the range of expression per cell varies between cell types, from 5655 receptors per cell in Th cells to 35 receptors per cell in adult hepatocytes. Another cytokine that was activated directly by IL-4 treatment was IL-6. Although mainly known for its role in inflammation, IL-6 is also involved in immune system regulation, cell proliferation, tissue regeneration, glucose metabolism, lipid metabolism, bone homeostasis, anti-inflammatory responses, and anti-apoptosis. IL-6 activation in disc cells averages about 15 times the mRNA level when stimulated with IL-4 [202].

IVD cells express a functional IL-4R and can respond to IL-4 to inhibit inflammation. These results suggest that incorporating local delivery of recombinant IL-4 to the disc would be a useful therapeutic strategy for treating patients with back pain, reducing inflammation and IDD [200]. IL-4 treatment helped to reduce basal mRNA levels of other inflammatory markers (IL-8 and IL-12), suggesting that IL-4 and low levels of IL-6 had an anti-inflammatory effect. To confirm that IL-4 has an anti-inflammatory effect on IVD cells, IVD cells were treated with lipopolysaccharide in the presence or absence of IL-4. IL-4 significantly reduced lipopolysaccharide-induced inflammatory gene expression (*CD68*, *IFNB*, *IL6,* and *IL8*) and the release of inflammatory cytokines (IL-6 and IL-8) [195]. IL-4 may upregulate opioid receptor expression, thereby influencing pain perception in IDD [203].

Increased expression of IL-4 and reduced expression of IFN-c indirectly demonstrate a higher tendency of Th to differentiate into Th1 during IDD [204]. Wang et al. [203] reported that IL-4 expression in IVD was significantly higher in lower-severity sciatica compared to higher-severity sciatica. Another study [205] found higher levels of IL-4 expression in tissue samples from patients with IVD degradation and herniation compared to autopsy IVD tissues (death due to trauma). Park et al. [206] compared subglottic extruded IVD with transligamentous extrusion or sequestration and found that IL-4 expression was significantly higher in the first group. Shamji et al. [164] found that IL-4 levels were higher in IVD hernia tissue samples than in degenerated IVD. Weber et al. [157] found that serum IL-4 levels were higher in patients with low back pain compared to age- and sex-matched healthy controls. Conversely, Capossela et al. [207] found lower levels of IL-4 expression in patients with chronic low back pain compared to healthy individuals without low back pain.

The study by Kedong et al. [200] showed a significant association of two SNVs (rs2243250 (590 C/T) and rs2070874 (33 C/T)) of the *IL4* gene with IDD in the Iranian population. In addition, SNV rs2243250 was associated with a reduction in postoperative pain in patients with IDD.

#### 3.2.2. Interleukin 6

IL-6 is a multifunctional pro-inflammatory cytokine that is secreted predominantly by monocytes and macrophages. It plays a key role in processes related to immunity and neuroinflammation [144]. This cytokine promotes the maturation of B cells into antibody-producing cells [208]. IL-6 stimulates the formation of osteoclasts and promotes bone resorption [209]. Signaling is activated by IL-6 via IL-6R and sIL-6R, which contain a signaling component (gp130). IL-6 binding to the IL-6R/gp130 complex primarily signals through the JAK/STAT, Ras, and PI3K pathways, and its function ranges from growth and differentiation of B and T cells to induction of acute phase proteins [104]. Many stimuli that activate IL-6 are associated with oxidative stress and damage to IVD cells [210]. IL-6 is involved in the pathogenesis of various autoimmune diseases such as rheumatoid arthritis and chronic inflammatory proliferative diseases [211].

Low- and high-affinity IL-6 receptors have been found in various tissues and organs, including B and T lymphocytes, endothelial cells, and IVDs. It has been shown that SNVs of the *IL6* gene are associated with discogenic lumbar pain. The number of receptors expressed on the cell surface varies depending on the cell type and averages about 1500 per cell, increasing with IVD hernias [2]. Studer et al. [108] found that IL-6 potentiates the catabolic effect of IL-1 and TNF-α on NP cells. Sainoh et al. [212] established the role of the IL-6 signaling cascade in enhancing the secretion of pain-associated proteins in the dorsal ganglion in an IVD animal model. IL-6-induced increase in TNF-α secretion was associated with apoptosis of dorsal spinal ganglion neurons [213]. Thus, IL-6 is a cytokine that affects the development of allodynia and hyperalgesia in a patient with IDD and, as a result, is a potential for analgesic therapy, which was confirmed using a selective inhibitor of IL-6 in the experiment. Clinically, high baseline IL-6 levels in patients undergoing IVD hernia repair were associated with poorer postoperative recovery [2]. Epidural administration of tocilizumab, an anti-IL-6-R monoclonal antibody, relieves radicular leg pain, numbness, and low back pain [157].

IL-6 is significantly elevated in both type 1 and type 2 diabetes and is a proven risk factor and independent predictor of type 2 diabetes [214]. Circulating IL-6 levels have been found to be elevated with high-fat meals, physical activity, and before/after surgery [215].

IL-6 is highly expressed in IDD, causing low back pain and exhibiting both pro-inflammatory and anti-inflammatory functions [100]. It regulates inflammatory responses by reducing levels of pro-inflammatory cytokines and activating anti-inflammatory molecules, including IL-1 receptor antagonist protein, TNF-soluble receptor, and extrahepatic protease inhibitors [216]. STAT inhibition attenuates the effects of IL-6 in IVD, so the IL-6/JAK/STAT3 pathway is a potential therapeutic target for the treatment of IDD [217]. IL-6 suppresses H2O2-induced IVD cell death by increasing inhibitory levels that are involved in cell apoptosis and aging [124]. Moreover, IL-6 promotes the expression of proteins involved in IVD, including COX-2 and MMP-13. High expression of IL-6 in degenerative rat and human IVDs has been shown [97].

Alkhatib et al. [114] demonstrated that damaged IVD had significantly increased levels of IL-6, IL-5, IL-8, and monocyte chemoattractant protein 2 (MCP-2) compared to intact IVD. These cytokines, measured in diseased and degenerated IVD, have been shown to elicit a range of deleterious responses, exacerbating the inflammatory microenvironment that is characteristic of IDD [218]. IL-6 expression levels were consistently higher in IVD protrusion compared to normal IVD tissue, regardless of IDD subtype. At the same time, IL-6 levels increased with increasing severity of IDD [98].

Genetic association studies have established an association between the risk of IDD and the G allele of two SNVs (rs1800795 and rs1800797) of the *IL6* gene. Moreover, the GGG haplotype had a highly significant association with the development of IDD [219].

Rigal et al. [99] published a meta-analysis and demonstrated that the SNV rs1800797 of the *IL6* gene could be a predictive biomarker for IDD. Other authors [100] explained the association between the SNVs of *IL6* and *IL10* genes and the risk of IDD. Two SNVs (rs1800795 and rs1800797) of the *IL6* gene are strongly associated with a predisposition to IDD. The G allele approximately 1.38 and 1.35 times, respectively, increases the risk of IDD compared to GC/CC or GA/AA genotypes [220]. SNVs of the *IL6* gene are strongly associated with IDD, and the G allele of two SNVs (rs1800795 and rs1800797) are genetic predictors of IDD. The T allele (13306435) and the C allele (rs2069849) of the *IL6* gene are associated with increased expression and high plasma levels of IL-6 in patients with IDD. However, other studies did not find a statistically significant association [32].

Inhibition of Stat-3 phosphorylation suppresses *IL6* gene expression and reduces its secretion. This mechanism engages healthy IVD cells, leading to a partial subsequent recovery (limited ability to regenerate), as aggrecan and COX-9, but not the alpha-2 chain of type IX collagen, increase [221].

#### 3.2.3. Interleukin 10 

IL-10, which is secreted by Th2 clones, belongs to the IL-10 family of cytokines, including IL-19, IL-20, IL-22, IL-24, and IL-26 [221]. IL-10, an anti-inflammatory cytokine, is a protective factor in various tissues including articular cartilage and IVD tissues. IL-10 modulates the function and differentiation of various immune cells such as B cells, NK cells, granulocytes, and some related cells [222]. IL-10 is an important anti-inflammatory cytokine that acts through a transmembrane receptor complex to regulate various immune cell functions such as attenuation of pro-inflammatory cytokines, antigen presentation and aberrant immune response, and enhancement of immune tolerance [223].

IL-10 enhances TNF degradation by regulating the p38 MAPK pathway. Yin et al. [224] suggested, that IL-10 can inhibit p38 MAPK activation and significantly reduce cell apoptosis. Activation of the p38 MAPK pathway may lead to excessive production of pro-inflammatory cytokines and decreased production of anti-inflammatory IL-10 [225]. However, inhibition of the p38 MAPK pathway can reduce the expression of endogenous IL-10 and suppress its anti-inflammatory activity [226].

Elevated plasma levels of IL-10 have been reported in patients with IDD [100]. In addition, IL-10 levels were elevated in models of rheumatoid arthritis and osteoarthritis. This cytokine has anti-inflammatory, anti-catabolic as well as anti-apoptotic effects on chondrocytes and is a potential target for the treatment of IDD [227].

A common characteristic of IVD and osteoarthritis is the degradation of the extracellular matrix by the regulation of MMP and other degrading enzymes to accelerate cellular apoptosis [104,228]. Clinically, IL-10 inhibits the catabolic effects of pro-inflammatory cytokines by suppressing MMP and pro-inflammatory COX-2 [229]. IL-10 antagonizes extracellular matrix-degrading enzymes and affects cartilage extracellular matrix gene expression triggered by pro-inflammatory cytokines such as TNF-α [230]. Behrendt reported that IL-10 significantly reduced the expression of ADAMTS-4, MMP-3, and MMP-13, which were strongly associated with extracellular matrix degradation, suggesting that IL-10 has a protective effect on chondrocytes [231]. Apoptosis contributes significantly to the pathogenesis of osteoarthritis and IDD [232]. IL-10 inhibits cell apoptosis by suppressing activated caspase-3 levels and bax/bcl-2 ratio to improve the osteoarthritis process. In addition, it inhibits TNF-α-induced mitochondria-dependent apoptosis by increasing bcl-2 levels and decreasing levels of cleaved caspase 3 [229,231].

De Waal et al. [233] demonstrated that IL-10 treatment can inhibit the production of IL-1α, IL-1β, IL-6, IL-8, TNF-α, GM-CSF (granulocyte macrophage colony-stimulating factor), and G-CSF (granulocyte colony-stimulating factor). Kühn et al. [234] confirmed the anti-inflammatory role of IL-10. IL-10 can suppress the pro-inflammatory activity of NK, monocytes, and macrophages, and also reduce the production of pro-inflammatory cytokines such as IL-12, TNF-α, and IFN-γ. It can also inhibit the synthesis of pro-inflammatory mediators and accelerate the degradation of their mRNA in neutrophils and NK cells by suppressing the NF-κB signaling pathway. IL-10 acts early in production, acting faster than TGF-β. There is an interaction between TGF-β and IL-10 as the combination of the two has a greater effect than either of them as a single agent. Cytokine mRNA expression and extracellular cytokine levels measured by fluorescence-activated cell sorting (FACS) assay showed that inflammatory cytokine expression in IDD can be blocked by exogenous TGF-β and IL-10, which may have a therapeutic effect in this disease. Either TGF-β or IL-10 alone suppressed the expression of inflammatory cytokines. In addition, their combined use resulted in a higher level of inhibition of TNF-α and IL-1β than TGF-β or IL-10 alone. There was a cumulative effect of their application [235].

Exogenous IL-10 facilitates IL-1β-induced degeneration of NP cells. In addition, IL-10 treatment increases the mRNA and expression of the *COL2A1* gene. However, a positive effect of IL-10 on collagen type X and SOX-9 mRNA expression and an increase in the level of aggrecan protein was not observed [236].

The *IL10* gene promoter is highly polymorphic. Three SNVs of the *IL10* gene localized in the promoter region are the most studied: substitutions -1082 G>A, 819 C>T, and -592 C>A. These SNVs may be associated with changes in bone mineral density and the development of osteoporosis. The GG genotype at -1082 C>T may be associated with higher IL-10 production. Carriers of the CC genotype at -592C>A had lower levels of IL-10 mRNA than carriers of the AA or AC genotypes, suggesting that the A allele may be a genetic predictor of IDD [237].

**Figure 3:**
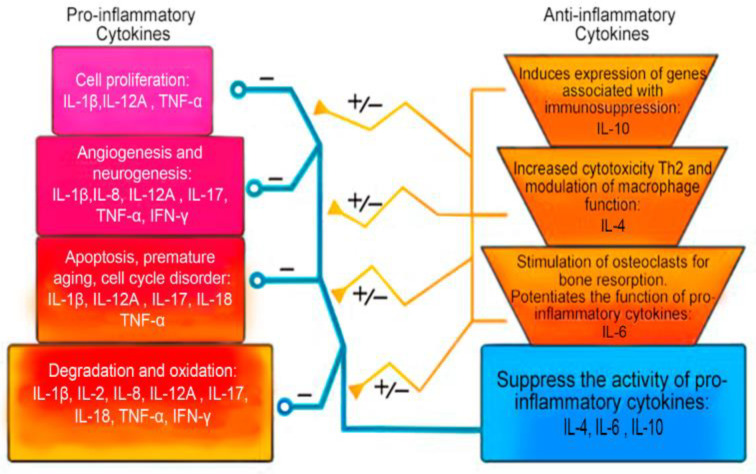
Mechanisms for modulating the effects of pro-inflammatory cytokines by anti-inflammatory cytokines in intervertebral disk degeneration. Note: IL—interleukin; TNF-α—tumor necrosis factor-alpha, IFN-γ—interferon-gamma; (−)—suppression of pro-inflammation cytokines effect; (+/−)—modulation of pro-inflammation cytokines effect.

A summary of the role of anti-inflammatory cytokines in IDD is presented in Table 5 and Figure 3.

## 4. Discussion

IDD is one of the main causes of back pain. This problem is also associated with a heavy economic burden, the prevalence of which increases with the age of the population [238]. The etiology of IDD is multifactorial, depends on age, cell-mediated processes of molecular degradation, and genetics, which is accelerated due to the effects of traumatic or prolonged mechanical factors. Thus, IDD is characterized by biochemical and cellular changes in the disc tissue [36,239,240]. Occupational habits, such as weightlifting, and lifestyle habits, such as not exercising and driving for long periods, contribute to the development and progression of IDD [241]. Smoking and injuries are also associated with the pathogenesis of IDD [242]. It is important that these various etiological factors can serve as primary initiating events that lead to abnormal production of cytokines and catabolic molecules by IVD cells [104]. However, it should be recognized that the mechanisms involved in the initiation, development, and progression of IDD are not clearly established and need further study.

A deeper knowledge of the role of inflammatory reactions in the complex spatial and temporal organization of cellular interactions and remodeling of the extracellular matrix of IVDs can play a crucial role in improving modern methods of IDD treatment, some of which still do not allow the achieving of the expected therapeutic response. Inflammation correlates with osteochondrosis, but its role in discogenic pain and hernia regression remains controversial [243]. During the pathological process of IDD development, both passive and active immune privileged barriers are damaged, and various mechanisms may be involved [93,244]. These mechanisms disrupt the immune balance of the IVD microenvironment. Multiple amplification of the lower cascades of the immune system can involve various specific and non-specific immune cells in the tissues of degenerating IVD, together with the cytokines that they secrete, aggravate the pathological condition, as well as hinder recovery, and cause acute and chronic back pain. At the same time, with an increase in the expression of growth factors and inflammatory cytokines in the autoimmune region of IVD and its microenvironment, neovascularization and neurogenesis are activated [245,246]. As IDD develops, elevated levels of pro-inflammatory cytokines accelerate this pathological process, increasing the degradation of aggrecan, as well as collagen, and contributing to phenotypic changes in IVD cells and their microenvironment [247]. Moreover, pro-inflammatory cytokines can induce IVD cell death and degradation of the intercellular matrix, thereby contributing to the further progression of IDD [104].

Thus, the inflammatory response may be involved in the onset of the disease, but it is also crucial for maintaining tissue homeostasis. In addition, with the optimal cytokine balance, it can contribute to the restoration/regeneration of IVD tissues. Pro-inflammatory and anti-inflammatory cytokines are important regulators of IDD.

Traditionally, the inflammatory response is mainly regarded as a negative and damaging mechanism that correlates with the progression of IDD. However, it remains unclear whether inflammation in general, and cytokine imbalance, in particular, are the cause or consequence of IDD and hernia formation. In order to reduce the rate of progression of the disease and the most complete (most possible in each specific clinical case) restoration of IVD function, a balanced inflammatory response is probably necessary, as previously proposed for other tissues of the human body [248,249].

**Figure 4:**
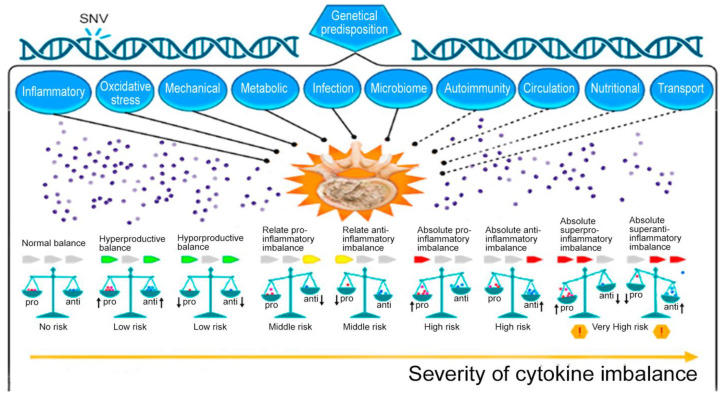
Potential role of normal and abnormal cytokine levels in the cytokine imbalance as a biomarker of intervertebral disk degeneration. Note: SNV—single-nucleotide variant; pro—pro-inflammation cytokines; anti—anti-inflammation cytokines. Note: solid line—well-studied mechanisms of IDD development; dotted line—insufficiently studied mechanisms of IDD development; up arrow—increased cytokine level; down arrow—decreased cytokine level; red exclamation point in a yellow hexagon is a dangerous situation.

The delicate balance between pro-inflammatory and anti-inflammatory cytokines determines the overall effect of the inflammatory response in patients with IDD. Disturbances in this balance can direct the protective immune response in an IDD patient toward chronic inflammation (pro-inflammatory) or towards healing (anti-inflammatory). Thus, an imbalance of cytokines may be beneficial for the IDD patient by initiating an inflammatory response in the NP, AF, and extracellular matrix. However, overproduction or underproduction of pro-inflammatory or anti-inflammatory endogenous mediators (cytokines) may actually be harmful and initiate the development of IDD (Figure 4).

Of great importance in children and young patients is the genetic predisposition, which determines the balance of pro-inflammatory and anti-inflammatory cytokines and, therefore, the predisposition to IDD [21].

Thus, IDD is mediated by abnormal production of pro-inflammatory and anti-inflammatory cytokines secreted by both NP and AF cells (fibrous cartilage tissue that contains NP), as well as macrophages, T cells, and neutrophils [250]. Cytokine imbalance causes a number of pathogenic reactions on the part of IVD cells, which can contribute to autophagy, aging, and apoptosis [251,252]. Cytokine imbalance may also be a critical factor in IDD. Recent studies have identified inflammatory mediators and signaling pathways as important factors in the onset and progression of IDD [253]. Inflammation mediated by immune cells increased in degenerated IVDs, and the decay products of these cells, such as IL-4, IL-6, IL-12, IFN-γ, and MMPs, led to a decrease in the number of NP cells and deterioration of the IVD microenvironment [254]. Prolonged inflammation recruits inflammatory cells that aggravate this situation. In addition, inflammatory mediators such as TNF-α and IL-1β induce the expression of pain-related factors such as nitric oxide, cyclooxygenase 2, and nerve growth factors that promote nerve ingrowth. All these factors together contribute to the occurrence of discogenic back pain.

An in-depth understanding of the contribution of cytokines and immune cells to IDD, inflammation, and nociception provides new potential targets for the treatment of this disease without interfering with the tissue repair program [104]. Since pro-inflammatory and anti-inflammatory cytokines form a complex regulatory network, it is reasonable to assume that the targeted action of a single cytokine may have a limited clinical effect in patients with IDD. Correction of cytokine imbalance in IDD requires the development of new therapeutic strategies that simultaneously target the specific actions of several key cytokines.

For example, therapeutic strategies targeting pro-inflammatory cytokines may be effective in the treatment of IDD. Pro-inflammatory cytokines are known to be critical in initiating an inflammatory response. However, their level in IVD could reach its absolute or relative peak before the clinical signs of IDD became apparent. In addition, therapy that blocks pro-inflammatory cytokines, paradoxically, can lead to an increase in the induced process of inflammation.

This narrative review provides new insight into the role of an imbalance between pro-inflammatory and anti-inflammatory cytokines in the pathogenesis of IDD and sets new targets for developing future therapeutic strategies in patients with IDD and low back pain.

## 5. Conclusions

This narrative review demonstrates that the problem of assessing the contribution of pro-inflammatory and anti-inflammatory cytokines to maintaining or changing the cytokine balance may become a new key to unlocking the mystery of IDD and developing new therapeutic strategies for the treatment of IDD, as well as acute and chronic discogenic back pain. In addition, the inconsistency of the results of previous studies on the role of pro-inflammatory and anti-inflammatory cytokines suggests that the biomarker of IDD is most likely not the serum level of one or more cytokines, but the cytokine balance. This supports the hypothesis that cytokine imbalance may be an important biomarker of IDD.

## Figures and Tables

**Table 1:** Hypotheses of intervertebral disc degeneration.

Hypothesis	Mechanism	References
Genetic	Congenital malformations IVD. Monogenic hereditary differentiated and undifferentiated connective tissue dysplasia. Genetic predisposition to impaired vitamin D metabolism ApaI (rs7975232), etc. Post-translational disorders (expression of zinc finger long non-coding RNA antisense 1). MicroRNAs as factors of degradation, inflammation, apoptosis, autophagy, or regulators of mechanosensory perception. Genetic predisposition to overproduction of pro-inflammatory cytokines, including SNVs of the genes: *IL1A* (rs1800587, rs2071375), *IL1B* (rs1143634), *IL4* (rs2243250 rs2070874); *IL-6* SNV in Exon 5 (rs13006435) other three SNVs of *IL-6* (rs1800797, rs1800796, rs1800795, rs13006435). Genetic predisposition to impaired synthesis of IVD components, including genes: *ACAN* (rs1042631, rs1516797), *COL1A1* (rs2075555, rs1007086, rs1800012, etc.), *COL9A2* (rs137853213), *COL9A3* (rs61734651), *COL9A1* (rs696990, rs7533552), *COL11A2* (rs1800587, rs1337185, rs1463035, rs2072915, rs9277933, rs2076311), *HAPLN1* (rs179851), *THBS2* (rs9406328), *COMP* (rs137852650), *CD36* (rs3173798, rs3211892), *CILP* (rs2033711); *ASPN* (rs373444, rs13301537), *GDF-5* (rs143383), etc. Genetic predisposition to increased oxidative processes in IDD: *MMP1* (rs1799750), *MMP2* (rs243865), *MMP3* (rs3025058), *MMP9* (rs17576), *PARK2* (rs926849), *RSMB9* (rs2187689, rs7767277), etc.	[12] [13,14] [15] [16] [17,18] [19,20] [21,22] [21,23]
Mechanical	Overweight and pathological obesity. Sedentary lifestyle (physical inactivity). Lifting weights, sharp turns, frequent bending, twisting, prolonged static load on the spine. Injury of the vertebral motor segment.	[24,25] [26] [26] [27]
Metabolic	Smoking. Hypoxia and high hemoglobin levels. Knockout of hypoxia-induced factor (HIF)-1alpha. High cholesterol and hyperlipidemia. Hyperglycemia. Hyperuricemia. Metabolic syndrome; Hypothyroidism.	[28] [28,29] [30] [31,32] [33] [25,34] [25,35] [36]
Circulation	Imbalance of bone homeostasis and osteoporosis. Violation of the blood supply (spasm of arterioles) of the end plate. Atherosclerosis of the arteries of the vertebral motor segment. Anemia, decompression sickness, Gaucher disease.	[37,38] [39] [32] [40]
Transport	Slow or insufficient outflow of lactate (acidification of the environment) IVD.	[41,42]
Oxidative stress	Activation of cathepsins in the acid environment of degenerating IVD. Activation of IVD matrix metalloproteinases. Homocysteine-induced oxidative stress and nucleus pulposus ferroptosis by increasing GPX4 methylation. Hemoglobin and heme-induced ferroptosis.	[43,44] [45,46] [47] [48]
Inflammatory	Absolute or relative overproduction of pro-inflammatory cytokines. Overexpression of ion channels of the TRP family (TRPV4). Overexpression of cytokine receptors. IL-6-induced ferroptosis. Abnormal activation of NLRP3 inflammasome (intracellular PRR). Adipokine resistin IVD degeneration associated with obesity.	[45,46] [49] [2] [50] [41,51] [24,52]
Autoimmunal	The *APOE* gene knockout and overexpression of catabolic cytokines in IVD. Autoimmune IVD degeneration due to FAS ligand hypo-expression.	[32] [53]
Microbiome	“Dysbacteriosis” (axis microbiome gut/skin/spine). Propionibacterium acnes and Staphylococcus epidermidis.	[54,55]
Infectional	Propionibacterium acnes, as a sluggish infection of IVD. Staphylococci (1% Staphylococcus epidermidis, 12% Staphylococcus auricularis, 12% Staphylococcus laminis, and 5% others).	[56,57] [58,59]
Nutritional	Autophagy. Increased nutritional requirements of IVD cells. Inadequate nutritional supply of IVD cells. Vitamin C deficiency in the elderly. Deficiency of proline, hydroxyproline. Vitamin D deficiency.	[60] [61,62] [63] [64] [65] [60,66]

Note: IVD—intervertebral disc; RNA—ribonucleic acid; SNVs—single nucleotide variants.

**Table 2:** Theories of cytokine role in intervertebral disc degeneration development.

Theory	Role of Cytokine	References
Theory of degradation of structures of the extracellular matrix of intervertebral disc	IL-1β, IL-6, IL-8, IL-17, IL-18, IL-21, IL-23, TNF-α, IFN-γ	[2,20,67,76,77,78,79,80,81,82,83,84,85,86,87,88,89,90,91,92,93,94,95,96,97,98,99,100]
Theory of oxidation	IL-1β, IL-6, IL-8, IL-17, IL-18, TNF-α, IFN-γ	[67,69,70,71,76,77,78,90,91,92,101,102,103,104,105,106,107,108]
Mechanical load theory	IL-1β, IL-6, IL-8, IL-17, TNF-α	[76,109,110,111,112,113,114,115]
Theory of programmed cell death	IL-1β, IL-6, IL-17, IL-18, TNF-α	[2,69,72,75,76,116,117,118,119,120,121,122,123,124]
Theory of cell proliferation	IL-1β, IL-17, TNF-α	[76,77,90,117,125,126,127,128,129,130,131]
Theory of premature aging	IL-1β, IL-6, IL-18, TNF-α	[39,73,74,76,77,124,132,133,134,135]
Autophagy theory	IL-1β, IL-17, TNF-α, IFN-β1	[71,72,90,133,136,137,138]
Theory of angiogenesis and neoinnervation	IL-1β, IL-17, TNF-α	[76,90,139,140,141,142]
Theory of hypoxia	IL-1β, TNF-α	[76]
Cell cycle disorder theory	IL-1β, IL-17, TNF-α	[77,143]

Note: IL-1β—interleukin 1 β; IL-6—interleukin 6; IL-8—interleukin 8; IL-17—interleukin 17; IL-18—interleukin 18; IL-21—interleukin 21; IL-23—interleukin 23; TNF-α—transforming growth factor alfa; IFN-γ—interferon gamma; IFN-β1—interferon beta 1.

**Table 3:** Pro-inflammatory and anti-inflammatory cytokines [144].

Pro-Inflammatory Cytokines	Anti-Inflammatory Cytokines
Interleukin 1 alpha (IL-1α) Interleukin 1 beta (IL1-β) Interleukin 6 (IL-6) Interleukin 8 (IL-8) Interleukin 11 (IL-11) Interleukin 12 (IL-12) Interleukin 17 (IL-17) Interleukin 18 (IL-18) Interleukin 20 (IL-20) Interleukin 33 (IL-33) Interferon gamma (IFN-γ) Tumor necrosis factor alpha (TNF-α) Transforming growth factor beta (TGF-β) Ciliary neurotrophic factor (CNTF) Granulocytic-macrophage colony-stimulating factor (GM-CSF) Leukemia inhibitory factor (LIF) Oncostatin M (OSM)	Interleukin 1 receptor antagonist (IL-1Ra) Interleukin 4 (IL-4) Interleukin 6 (IL-6) Interleukin 10 (IL-10) Interleukin 11 (IL-11) Interleukin 13 (IL-13) Interleukin-18-binding protein (IL1-8BP) Transforming growth factor beta (TGF-β)

**Table 4:** Role of pro-inflammatory cytokines in intervertebral disk degeneration.

Cytokine	Gene: OMIM Number	Role in Intervertebral Disk	Clinical Role in IDD	References
IL-1β	*IL1B*: 147720	Initiation of inflammatory, oxidative, degenerative, apoptotic cascades. Association with premature aging and cell growth arrest. Over-expression of vascular endothelial growth factor, NGF, and BDNF.	+++	[67,69,73,75,76,77,102,118,132,141,151]
IL-2	*IL2*: 147680	Growth factor. Initiation of the inflammatory and degenerative cascade.	+	[155,156]
IL-8	*CXCL8:* 146930	Increased migration (potent chemokine) of neutrophils, T cells, and monocytes, whose enzymes produce free oxygen radicals. Indirect increase in oxidative stress, which can lead to IVD cell death. Involvement in the pathogenesis of acute neuropathic pain.	+	[85,86,87,88,89,106,107,109,110,111,158]
IL-12A	*IL12A*: 161560	Stimulation of proliferation. Activation and increase in the cytotoxicity of NK cells and T cells. Stimulation of differentiation in Th1. Induction of IFN-γ and TNF-α secretion, synergism with pro-inflammatory cytokines with IL-18.	++	[141,144]
IL-17	*IL17A*: 603149	Initiation of the inflammatory and degenerative cascade. Association with cell growth arrest. Stimulation of angiogenesis.	+++	[68,72,90,91,92,93,94,96,113,115,165,167,172]
IL-18	*IL18:* 600953	Initiation of the inflammatory and degenerative cascade (IFN-γ activation). Initiation of the apoptotic and oxidative cascade. Association with premature aging of IVD cells.	+++	[70,83,105,121,122,123]
TNF-α	*TNF:* 191160	Initiation of inflammatory, apoptotic, oxidation, and degenerative cascades. Association with premature aging and cell growth arrest. Autophagy promoter While TNF-α signaling via TNFR2 is anti-inflammatory and cytoprotective, resulting in the induction of proliferation, differentiation, angiogenesis, and tissue repair.	+++	[71,74,76,77,79,80,104,116,117,126,127,130,133,135,144]
IFN-γ	*IFNG:* 147570	Initiation of the inflammatory and degenerative cascade in IVD cells. Involvement in the pathogenesis of acute neuropathic pain in IDD.	+++	[2,156,164,191,192]

Note: IDD—intervertebral disc degeneration; (+)—questionable prognostic role in the development of IDD; (++)—moderate prognostic role in the development of IDD; (+++)—significant prognostic role in the development of IDD; BDNF—brain-derived neurotrophic factor; IFN-γ—interferon-gamma; IL-12—interleukin 12; IL-17—interleukin 17; IL-18—interleukin 18; IL-1β—interleukin 1 β; IL-2—interleukin 2; IL-8—interleukin 8; NGF—nerve growth factor; NK cells—natural killer cells; T cells—T-lymphocytes; Th1—type 1 helper T cells; TNFR2—tumor necrosis factor receptor 2; TNF-α—tumor necrosis factor-alpha.

**Table 5:** Role of anti-inflammatory cytokines in intervertebral disk degeneration.

Cytokine	Gene: OMIM Number	Role in Intervertebral Disk	Clinical Role in IDD	References
IL-4	*IL4:* 147780	Initiation of Th differentiation into Th2 lymphocytes. Increased Th2 cytotoxicity. Modulation of the function of macrophage cells. Decreased cytotoxicity. Inhibition of LPS, IFN gamma, and induction of TNF-α, IL-1α pathways of degeneration. Induction of production of IL-1β and TNF-α. Stimulation of IL-6 activation and participation together with it in anti-inflammatory, antioxidant activity. Decreased LPS-induced expression of IL-8, IL-12.	+++	[157,164,194,195,196,199,200,202,203,205,207]
IL-6	*IL6:* 147620	A key role in the processes associated with immunity and inflammation. Potentiates the inflammatory, degenerative and oxidative cascade. May act as an anti-inflammatory cytokine. Able to stimulate osteoclasts and bone resorption. Suppresses H_2_O_2_-associated premature aging and apoptosis.	+++	[2,32,97,98,99,100,104,108,114,124,157,210,212,213,215]
IL-10	*IL10:* 124092	Initiation of cellular effects through canonical JAK/STAT, which includes JAK1 and STAT3. Induction of expression of genes associated with immunosuppression. Providing antigens and enhancing immune tolerance. Anti-inflammatory, anti-catabolic, and anti-apoptotic action. Decreased production of IL-1α, IL-1β, IL-6, IL-8, IL-12, TNF-α, IFN-γ, GM-GSF and GCSF.	+++	[104,224,225,227,228,229,230,231,235,236]

Note: IDD—intervertebral disc degeneration; (+++)—significant prognostic role in the development of IDD; IL-4—interleukin 4; IL-6—interleukin 6; IL-10—interleukin 10; Th—helper T cells; LPS—lipopolysaccharide; IFN—interferon; TNF-α—tumor necrosis factor-alpha; IL-8—interleukin 8; IL-12—interleukin 12; JAK—Janus kinase; JAK1—Janus kinase 1; STAT—signal transducer and activator of transcription; STAT3—signal transducer and activator of transcription 3; GM-GSF—granulocyte-macrophage colony-stimulating factor; GCSF—granulocyte colony-stimulating factor.

## Data Availability

Not applicable.
